# Gelation and Orientation Dynamics Induced by Contact of Protein Solution with Transglutaminase Solution

**DOI:** 10.3390/gels9060478

**Published:** 2023-06-12

**Authors:** Kasumi Kakinoki, Ryuta Kurasawa, Yasuyuki Maki, Toshiaki Dobashi, Takao Yamamoto

**Affiliations:** 1 Division of Molecular Science, Graduate School of Science and Technology, Gunma University, Kiryu 376-8515, Japan; 2 Department of Chemistry, Faculty of Science, Kyushu University, Fukuoka 819-0395, Japan; 3 Division of Pure and Applied Science, Graduate School of Science and Technology, Gunma University, Kiryu 376-8515, Japan

**Keywords:** gel growth dynamics, anisotropic gel, liquid–liquid contact, free-energy-limited process, diffusion-limited process, protein, enzyme

## Abstract

Gel growth induced by contact of polymer solutions with crosslinker solutions yields an emerging class of anisotropic materials with many potential applications. Here, we report the case of a study on the dynamics in forming anisotropic gels using this approach with an enzyme as a trigger of gelation and gelatin as the polymer. Unlike the previously studied cases of gelation, the isotropic gelation was followed by gel polymer orientation after a lag time. The isotropic gelation dynamics did not depend on concentrations of the polymer turning into gel and of the enzyme inducing gelation, whereas, for the anisotropic gelation, the square of the gel thickness was a linear function of the elapsed time, and the slope increased with polymer concentration. The gelation dynamics of the present system was explained by a combination of diffusion-limited gelation followed by free-energy-limited orientation of polymer molecules.

## 1. Introduction

Most industrial gels of our surroundings are isotropic, since they are prepared from solutions by mixing them with crosslinkers homogeneously, lowering or raising the temperature, exposing them to high-energy electromagnetic waves, etc. according to the principle of maximum entropy. The gelation dynamics has been studied classically according to the tree model, and the time development of molecular weight distribution was discussed [1,2,3]. More recently, using the analogy between gelation and critical phenomena, the sol–gel transition was analyzed using a set of scaled equations [4,5]. Furthermore, many comprehensive reviews or books are known for specific types of gelation, such as thermo-reversible gelation [6] and gelation of colloidal dispersion [7]. 

On the other hand, when gelation starts from a point or a surface of a solution, a gel region is developed in the solution, the degree of gelation is characterized by the gel volume, and its time development needs to be clarified. A typical example is illustrated by dripping a drop of concentrated polymer solution into a crosslinker solution, where the crosslinking reaction occurs at the spherical surface of the drop to instantaneously make a thin gel membrane, and the diffusion of crosslinkers through it thickens the gel. As early as the 1950s, birefringence was observed for gels prepared using such a system via diffusion of multivalent ions into a polysaccharide solution [8]. A gradient of polymer concentration was observed for the system with polymer solutions in contact with multivalent ions through a dialysis membrane [9,10]. Anisotropic gels are induced by the diffusion of various types of gel inducers such as crosslinkers and pH adjusters into polymer solutions [11,12]. The inhomogeneity is often coupled with other mechanisms such as spinodal decomposition [13,14], Ostwald ripening [15], and shrinking [16,17] to form more complex structures. Molecular orientation in the anisotropic gels induced by diffusion was carefully examined by X-ray and light scattering, as well as wave plates, by Maki and Furusawa et al. and other groups [14,16,17,18,19,20]. Gels having such a characteristic structure were applied to prepare artificial or engineered tissues and organs [21,22,23,24,25], carcinogen adsorbents [26], and mechano-optical sensors [27].

The time course of gel growth by diffusion from a surface was explained by focusing the motion of the gel front line, i.e., the “moving boundary picture (MB)”, proposed by Yamamoto et al. [28,29,30,31]. The main conclusion of the theory is that the dynamics is expressed by scaled equations that are categorized into several types depending on the gelation mechanism and geometrical condition [11,12]. Since gelation dynamics affects the characteristics of the resultant gel, it is very important to clarify the dynamics of not yet examined gelations. 

The gels that make up parts of living organisms such as blood vessels, skin, and even blood coagulants are grown from the surface of endothelial cells on blood vessel walls, skin cells, etc., resulting in a structure with more or less anisotropic nature. Transglutaminase is an enzyme that catalyzes crosslinking reactions of proteins, which is significant both physiologically and industrially [32,33,34,35,36]. A type of transglutaminase, Factor XIII, plays an essential role in the last stage of blood coagulation to cross-bridge the physically bonded fibrin gel called the soft clot to form the covalently bonded hard clot [37,38]. Other types of transglutaminase have physiological functions such as cell adhesion, wound healing, apoptosis, and extracellular matrix development [39]. Participation of tissue transglutaminase in the development of neurodegenerative disorders such as Alzheimer’s disease has been intensively discussed [40]. Transglutaminase is also used in the food industry and molecular gastronomy to meld new textures with existing tastes [41]. Therefore, the gelation induced by transglutaminase has the possibility of extensive applications. However, the dynamics of gelation induced by transglutaminase in these phenomena has not yet been clarified. 

Here we report an observed gel growth from an interface between gelatin solution and microbial transglutaminase solution as a model case. When transglutaminase solution was put on the isotropic gel, interestingly, an isotropic gelation was observed and followed by gel polymer orientation after a lag time for this system. A theoretical analysis was conducted for the gelation and orientation dynamics of this system on the basis of the MB picture.

## 2. Theory

The mechanism of gelation induced by enzymes has a significant feature, different from that induced by crosslinkers. In the former, enzymes are not consumed, whereas, in the latter, crosslinkers are consumed during the gelation. Fortunately, the feature does not make the derivation of the gelation dynamics theory difficult. For the gelation induced by enzymes, the MB picture is also applicable. Thus, we can construct the dynamics of the gelation induced by enzymes on the basis of the MB picture and clarify the essential properties of the dynamics.

The experiment shows that the time courses of the isotropic gelation and of the anisotropic gelation are different. The difference requires introducing additional dynamics. Accordingly, we introduce relaxation dynamics based on the free-energy principle in which the state of the system develops to the lower free-energy state. The theory explaining the observed results is constructed by assuming that (i) the isotropic gelation occurs by diffusion of enzymes, followed by (ii) transforming the isotropic gel into the anisotropic gel by the free-energy principle [42]. The isotropic gelation process is expressed by the MB picture, and the dynamics from the isotropic gel to the anisotropic gel is described by the Ginzburg–Landau (GL) equation [43,44,45]. 

### 2.1. Gelation Dynamics by a Contact of Protein Solution with Transglutaminase Solution

The characteristic feature of the enzyme reaction is that the enzyme is repeatedly used for the reaction (not consumed in the reaction), and the enzyme reaction is much faster than the diffusion of enzymes (no lag time is needed for gel formation). Taking into account this feature, we can derive the gelation dynamics on the basis of the MB picture.

The feature requires that the protein solution in the region where transglutaminase molecules are present quickly turns into a gel without transglutaminase consumption. Therefore, the gel front line is equivalent to that of the protein solution region into which transglutaminase molecules diffuse from transglutaminase solution. Let us call the region the diffused region. Hence, the gel region is the diffused region. Since the diffusion process of transglutaminase is expected to be very slow, the growth dynamics of the diffused region is derived on the basis of the MB picture.

**Figure 1:**
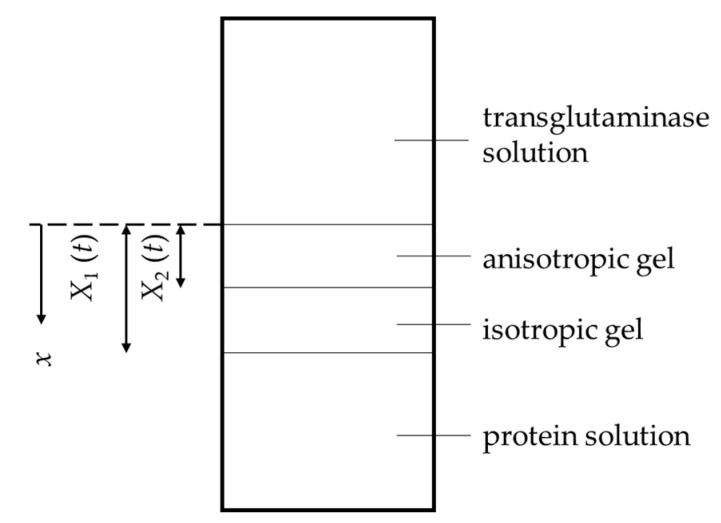
Illustration of anisotropic gel formation and notations. The origin of the *x*-axis is at the position of the initial boundary between the protein solution and the transglutaminase solution. *X*_1_ (*t*) and *X*_2_ (*t*) are the distances from the origin to the isotropic gel front line and to the anisotropic gel front line, respectively, at time *t*.

We take the origin *x* = 0 at the position of the initial boundary between the protein solution and the transglutaminase solution, as shown in Figure 1. Let us define the flux of transglutaminase molecules in the diffused region, the unit vector in the *x*-direction, and the concentration of transglutaminase solution as $\vec{j}$, ${\vec{e}}_{x}$, and $\rho_{s}$ (=*C*_t_). We also denote the position of the front line of the diffused region as *x* = *X*_1_. The flux of transglutaminase molecules $\vec{j}$ is expressed as

(1) $$\vec{j}\left(x\right)=j\left(x\right){\vec{e}}_{x}.$$

Note that $0\leq x\leq X_{1}$.

According to Fick’s law, we have

(2) $$j\left(x\right)=-k\rho \left(x\right)\frac{\partial \mu \left(x\right)}{\partial x},$$

where $\rho \left(x\right)$ and μ(x) are the concentration and the chemical potential of transglutaminase, respectively, at *x* in the diffused region, and *k* is the mobility of transglutaminase. Assuming *j* as the steady flow, we have

(3) $$div\vec{j}=\frac{\partial j}{\partial x}=0.$$

The equation gives

(4) $$j\left(x\right)=C,$$

where *C* is a constant. Using Equation (2), we can rewrite Equation (4) as

(5) $$-k\rho \frac{\partial \mu }{\partial x}=C.$$

The left-hand side of Equation (5) is expressed in terms of free energy per unit volume *f* satisfying μ=∂f/∂ρ or partial pressure g of inflow transglutaminase as

(6) $$\rho \frac{\partial \mu }{\partial x}=\frac{\partial }{\partial x}\left(\rho \mu \right)-\frac{\partial f}{\partial x}=\frac{\partial }{\partial x}\left(\rho \mu -f\right)=\frac{\partial g}{\partial x}.$$

Then, Equation (5) reduces to

(7) $$-k\frac{\partial g}{\partial x}=C.$$

Integrating both sides of the above equation from x=0 to $x=X_{1}$, we have $k\left[g\left(X_{1}\right)-g(0)\right]=CX_{1}$. Therefore, the constant C is given by

(8) $$C=-k\frac{g\left(X_{1}\right)-g\left(0\right)}{X_{1}}.$$

The flux is obtained as

(9) $$j\left(x\right)=-k\frac{g\left(X_{1}\right)-g\left(0\right)}{X_{1}},$$

and it is independent of the position in the diffused region.

Using the low-density limit approximation for transglutaminase molecules in the diffused region, we have

(10) $$\mu =k_{B}T\ln\rho ,$$

and

(11) $$g\left(x\right)=k_{B}T\rho \left(x\right).$$

From Equations (7)–(9), we have

(12) $$\frac{\partial \rho }{\partial x}=\frac{1}{k_{B}T}\frac{g\left(X_{1}\right)-g\left(0\right)}{X_{1}}.$$

Since the volume of the enzyme solution is large, the concentration of the enzyme solution $\rho_{s}$ can be regarded as constant independent of the elapsed time. The transglutaminase molecules do not reach the sol region $x>X_{1}$. Therefore, we have the boundary condition, $\rho \left(0\right)=\rho_{s}$ and $\rho \left(X_{1}\right)=0$, where

(13) $$\left\{\begin{array}{c} g\left(X_{1}\right)=k_{B}T\rho \left(X_{1}\right)=0\\ g\left(0\right)=k_{B}T\rho \left(0\right)=k_{B}T\rho_{s} \end{array}\right..$$

Then, Equation (12) reduces to

(14) $$\frac{\partial \rho }{\partial x}=-\frac{\rho_{s}}{X_{1}}.$$

Integrating both sides and using the boundary condition Equation (13), we have the concentration of transglutaminase as

(15) $$\rho \left(x\right)=\rho_{s}\left(1-\frac{x}{X_{1}}\right).$$

The number of transglutaminase molecules in the diffused region ${n(X}_{1})$ is obtained as

(16) $$n\left(X_{1}\right)=A\int_{0}^{X_{1}}\rho \left(x\right)dx=\frac{1}{2}\rho_{s}X_{1}A,$$

where A is the cross-sectional area of the vessel.

The inflow of transglutaminase molecules increases the number of transglutaminase molecules in the diffused region. The increase of transglutaminase molecules moves the front line of the diffused region away from the boundary between the transglutaminase solution and the diffused region. Hence, we can construct the following balance equation for the transglutaminase molecule number at the front line of the diffused region:

(17) $$j\left(x\right)Adt=dn\left(X_{1}\right)=\frac{dn\left(X_{1}\right)}{dX_{1}}dX_{1}.$$

Using Equations (9) and (13), we have transglutaminase flux independent of the position x as

(18) $$j\left(x\right)=-k\frac{g\left(X_{1}\right)-g\left(0\right)}{X_{1}}=kk_{B}T\frac{\rho_{s}}{X_{1}}.$$

Using Equations (16) and (18), we can rewrite Equation (17) as

(19) $$kk_{B}T\frac{\rho_{s}}{X_{1}}Adt=\frac{1}{2}\rho_{s}AdX_{1}.$$

The differential Equation (19) reduces to the time development equation of the front line,

(20) $$t=\frac{1}{K}X_{1}^{2},$$

with a constant

(21) $$K=4kk_{B}T.$$

Equation (20) can be regarded as the dynamics of the gel front line since the front line of the diffused region is equivalent to the gel front line. The dynamics given by Equation (20) is independent of transglutaminase concentration in the transglutaminase solution and the protein concentration. The transglutaminase concentration independence is the significant feature of the gelation by enzymes predicted by the MB picture.

### 2.2. Orientation Dynamics Based on the Free-Energy Principle

In the gelation induced by transglutaminase, the anisotropy appears, followed by gelation with a lag time. The anisotropy is induced by orientation of the gel polymer immersed in the transglutaminase solution. The fact that the anisotropy appears after the lag time shows that the rate-limiting process of the isotropic gelation dynamics of the gelatin solution and that of the orientation dynamics of the gelatin gel are different. Since the orientation dynamics is observed on a macroscopic space–time scale, the orientation process is expected to be a relaxation phenomenon driven by thermodynamic forces. Since the orientation occurs in an isothermal condition, the driving force is the difference in free energy between the isotropic and anisotropic gel phases. Hence, the orientation dynamics is free-energy-limited.

**Figure 2:**
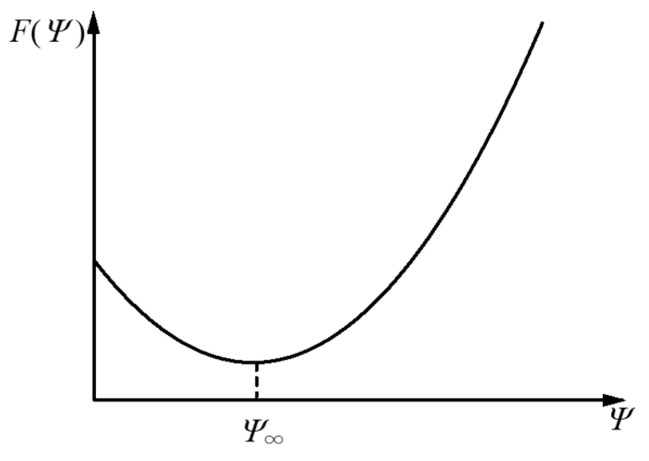
Illustration of free energy as a function of order parameter expressing the degree of orientation.

In order to discuss the dynamics of appearance of anisotropy, let us introduce the “order parameter” Ψ expressing the magnitude of anisotropy (the gel is isotropic when the value of Ψ in equilibrium is zero, whereas it is anisotropic when the value is finite); we assume that the change from the isotropic state to the anisotropic state (the orientation of the gel polymer) requires no additional in- and outflow of materials [42]. The free energy under fixed value Ψ of the degree of orientation is written as F(Ψ). The value of the order parameter after enough time has elapsed is denoted as $\Psi_{\infty }$. The enzyme influx first forms an isotropic gel (Ψ=0); then, the gelatin gel relaxes from an isotropic structure with Ψ=0 to an anisotropic structure with $\Psi =\Psi_{\infty }>0$ according to the second law of thermodynamics because $F\left(0\right)>F(\Psi_{\infty })$ (see Figure 2).

It is well known that this type of dynamics can be expressed by the time-dependent GL type equation [43,44,45] with respect to the time-dependent order parameter Ψ(t) at time t:

(22) $$\frac{\partial \Psi }{\partial t}=-\Gamma \frac{\partial F}{\partial \Psi }.$$

where F=F(Ψ) is the free energy per unit volume for the gel with anisotropy expressed by the order parameter Ψ expressing the magnitude of birefringence of the gel, and Γ is the kinetic coefficient having a positive value. The function F is expected to have a minimum at the finite value $\Psi_{\infty }$ of Ψ as shown in Figure 2. The free energy in the isotropic state with Ψ=0 is higher than that in the anisotropic state with $\Psi =\Psi_{\infty }$. The difference induces the time-development of the order parameter Ψ from zero to $\Psi_{\infty }$.

The value of $\Psi_{\infty }$ minimizing the free energy F generally depends on the concentration ϕ (=*C*_g_) of the gel network and ρ of transglutaminase. It expresses the equilibrium state of the protein gel with protein concentration ϕ immersed in the transglutaminase solution with transglutaminase concentration ρ. Let us adopt a third assumption: (iii) the protein gel is anisotropic when ρ is larger than a threshold transglutaminase concentration $\rho_{f}(\phi )$ depending on protein concentration ϕ, followed by assumptions (i) and (ii). The third assumption mathematically means that the value of $\Psi_{\infty }=\Psi_{\infty }(\phi ,\rho )$ is finite when ρ is larger than the threshold concentration.

It is difficult to derive the free-energy function form F=F(Ψ). However, the outline of the dynamics is obtained by the quadratic function approximation around $\Psi =\Psi_{\infty }$:

(23) $$F\left(\Psi \right)≅F_{\infty }+\frac{1}{2}B{\left(\Psi -\Psi_{\infty }\right)}^{2},$$

where $F_{\infty }=F(\Psi_{\infty })$ and $B=\partial^{2}F(\Psi_{\infty })/\partial \Psi^{2}$. Note that $\partial F(\Psi_{\infty })/\partial \Psi =0$. Therefore, Equation (22) is rewritten as

(24) $$\frac{\partial \Psi }{\partial t}=-\Gamma B\left(\Psi -\Psi_{\infty }\right).$$

Here, note that $\Psi_{\infty }$ depends on the elapsed time t since ρ increases by the transglutaminase flow $\vec{j}$.

Let us denote the starting time (the gelation time) at x as $t_{g}(x)$. Then, the initial condition for Equation (24) is given by

(25) $$\Psi \left(t_{g}\left(x\right)\right)=0.$$

Since the gel front position $X_{1}$ at time t is given by Equation (20), the gelation time at x is given by

(26) $$t_{g}\left(x\right)=\frac{1}{K}x^{2}.$$

Taking the time dependence of $\Psi_{\infty }$ (=$\Psi_{\infty }\left(\phi ,\rho \left(x,t\right)\right)=\Psi_{\infty }(t)$) into account, we solve Equation (24) on the basis of the initial condition and obtain the solution

(27) $$\Psi \left(t\right)=e^{-t/\tau }\frac{1}{\tau }\int_{t_{g}(x)}^{t}\Psi_{\infty }(t^{\prime })e^{t^{\prime }/\tau }dt{}^{\prime },$$

with

(28) $$\tau =\frac{1}{\Gamma B}.$$

From Equations (15) and (20), ρ(x,t) is given by

(29) $$\rho \left(x,t\right)=\left\{\begin{array}{cc} \rho_{s}\left(1-\frac{x}{\sqrt{Kt}}\right), & x\leq \sqrt{Kt}\\ 0, & x>\sqrt{Kt} \end{array}\right..$$

Since it is difficult to derive the explicit function form of $\Psi_{\infty }\left(\phi ,\rho \left(x,t\right)\right)$, we adopt the simplest expression for $\Psi_{\infty }\left(\phi ,\rho \left(x,t\right)\right)$ satisfying assumption (iii) as

(30) $$\Psi_{\infty }\left(\phi ,\rho \left(x,t\right)\right)=\left\{\begin{array}{cc} 0 & \left(\rho \left(x,t\right)<\rho_{f}\left(\phi \right)\right)\\ \Psi_{\infty ,0} & \left(\rho \left(x,t\right)>\rho_{f}\left(\phi \right)\right) \end{array}\right.,$$

where $\Psi_{\infty ,0}$ is a positive constant.

The orientation starting time $t_{a}=t_{a}(\phi ,x)$ at which transglutaminase concentration reaches the threshold concentration is estimated as

(31) $$\rho \left(x,t_{a}\right)=\rho_{f}\left(\phi \right),$$

and it is expressed as

(32) $$t_{a}\left(\phi ,x\right)=\frac{x^{2}}{{\left(1-\frac{\rho_{f}(\phi )}{\rho_{s}}\right)}^{2}K}=\frac{1}{{\left(1-\frac{\rho_{f}\left(\phi \right)}{\rho_{s}}\right)}^{2}}t_{g}\left(x\right).$$

Using Equation (32), we can rewrite the solution Equation (27) as

(33) $$\Psi =\Psi \left(x,t\right)=\left\{\begin{array}{cc} 0, & t<t_{a}(\phi ,x)\\ \Psi_{\infty ,0}\left(1-e^{-\left(t-t_{a}\left(\phi ,x\right)\right)/\tau }\right), & t\geq t_{a}(\phi ,x) \end{array}\right..$$

Note that the time development of the order parameter depends on the position x in the gel layer through the position dependence of the orientation starting time.

The characteristic time of the relaxation behavior Equation (33) is τ, and the relaxation is expected to be completed after the characteristic time. Then, the time $t_{ag}$ at which the orientation is completed is estimated as $t_{ag}-t_{a}≅\tau$ and is given by

(34) $$t_{ag}=t_{\mathrm{ag}}\left(x\right)=t_{a}\left(x\right)+\tau =\frac{x^{2}}{{\left(1-\frac{\rho_{f}(\phi )}{\rho_{s}}\right)}^{2}K}+\tau =\frac{1}{K^{\prime }}x^{2}+\tau ,$$

where

(35) $$K^{\prime }={\left(1-\frac{\rho_{f}}{\rho_{s}}\right)}^{2}K.$$

From Equation (34), the relationship between the front line $X_{2}$ of the anisotropic gel and the elapsed time t is obtained as

(36) $$t=t_{\mathrm{ag}}\left(X_{2}^{2}\right)=\frac{1}{K{}^{\prime }}X_{2}^{2}+\tau .$$

Equations (20) and (36) show that the elapsed time is expressed by a linear function of the square of the gel thickness both for the isotropic gel and for the anisotropic gel. The equation expressing the anisotropic-gel front line dynamics has the “lag time” τ and the slope $1/K{}^{\prime }$ depending on protein concentration and transglutaminase concentration, whereas that expressing the isotropic gel front line dynamics has no lag time and a “universal” slope.

## 3. Results and Discussion

### 3.1. Experimental Results and Data Fitting

**Figure 3:**
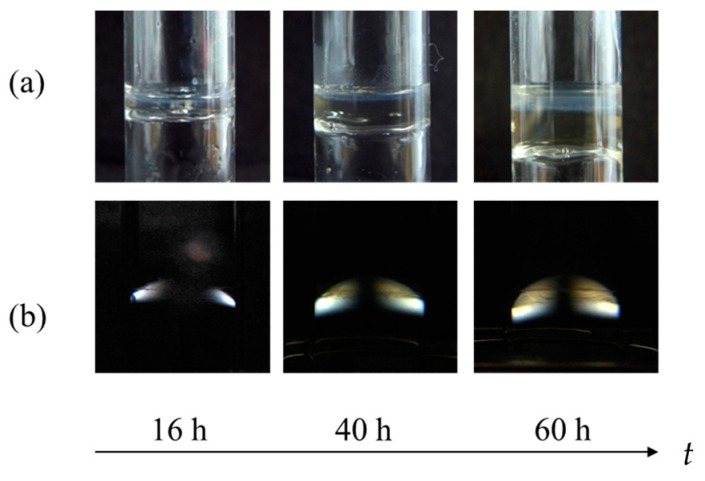
Time course of anisotropic gelation observed under natural light (**a**) and crossed nicols (**b**) at *C*_g_ = 20 wt.% and *C*_t_ = 0.25 wt.%.

**Figure 4:**
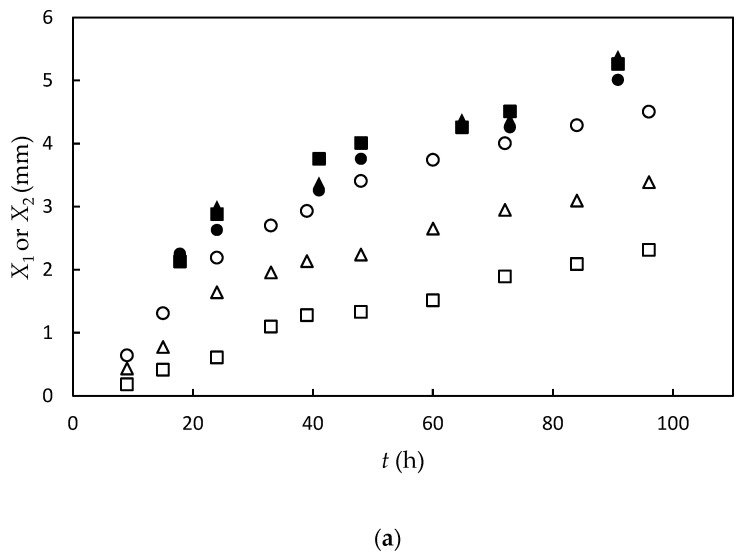

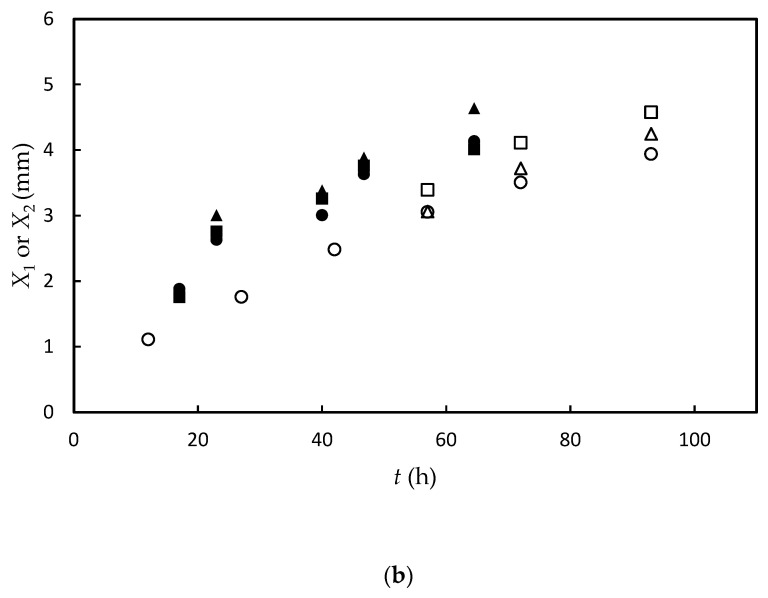
The thickness of gel layer *X*_1_ (closed symbol) and orientation layer *X*_2_ (open symbol) as a function of time at constant transglutaminase concentration of *C*_t_ = 0.25 wt% at various gelatin concentrations *C*_g_ = 10 wt.% (square), 15 wt.% (triangle), and 25 wt.% (circle) (**a**), and at constant gelatin concentration of *C*_g_ = 20 wt.% at various transglutaminase concentrations *C*_t_ = 0.05 wt.% (square), 0.1 wt.% (triangle), and 0.25 wt.% (circle) (**b**).

Figure 3a shows the photographs of the gel part observed under natural light after both upper and lower solutions were removed. Figure 3b shows the growth of the birefringent layer with time. Figure 4a,b show the time course of the average thickness of the gel layer *X*_1_ and the birefringent layer *X*_2_ observed in Series 1 for constant transglutaminase concentration and various gelatin concentrations, and Series 2 for constant gelatin concentration and various transglutaminase concentrations, respectively. The experiments show that the gelation dynamics does not depend on concentrations of gelatin and transglutaminase, whereas the orientation dynamics depends on concentrations of both gelatin and transglutaminase.

**Figure 5:**
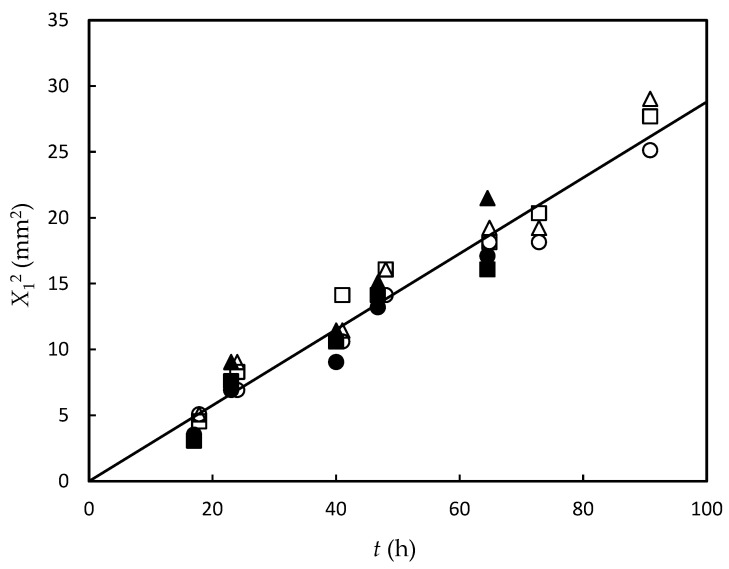
Proportional relationship between square of gel layer thickness and elapsed time for samples with concentrations of (*C*_g_, *C*_t_); (10 wt.%, 0.25 wt.%) (open square), (15 wt.%, 0.25 wt.%) (open triangle), (25 wt.%, 0.25 wt.%) (open circle), (20 wt.%, 0.05 wt.%) (closed square), (20 wt.%, 0.1 wt.%) (closed triangle), and (20 wt.%, 0.25 wt.%) (closed circle). The line is obtained by fitting the data to Equation (20), where *K* = 0.288 ${mm}^{2}\cdot h^{-1}$.

Figure 5 shows the plot according to Equation (20). All data points for different *C*_g_ and *C*_t_ are on a line. Hence, the gelation dynamics does not depend on the concentrations of transglutaminase and gelatin. The universal behavior asserts the validity of the theoretical predictions in the previous section. From the slope, *K* was determined as 0.288 ${mm}^{2}\cdot h^{-1}$. From Fick’s law (Equation (2)) and the low-density limit approximation shown by Equation (10), the diffusion coefficient of transglutaminase D is estimated as $D={kk}_{B}T=K/4≅2.0\times 10^{-11}$ m^2^·s^−1^.

**Figure 6:**
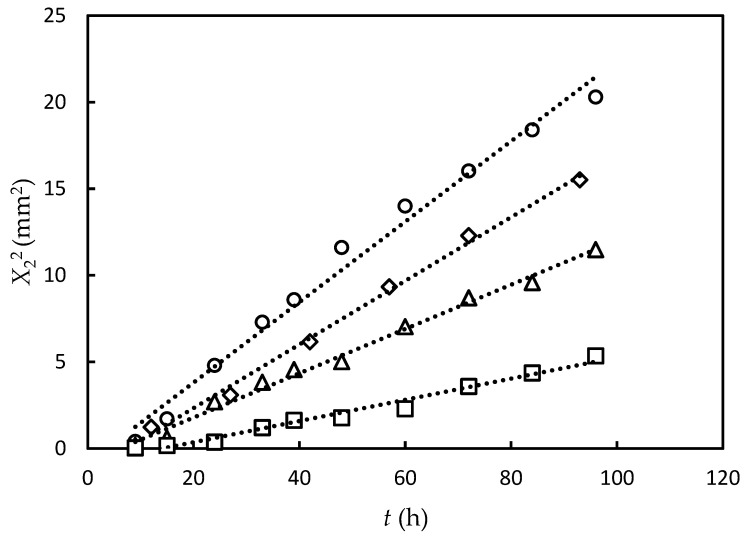
Square of thickness of orientation layer vs. time for samples with concentrations (*C*_g_, *C*_t_); (10 wt.%, 0.25 wt.%) (square), (15 wt.%, 0.25 wt.%) (triangle), (20 wt.%, 0.25 wt.%) (diamond), and (25 wt.%, 0.25 wt.%) (circle). The dashed lines are a guide to the eyes.

As shown in Figure 6, we plotted the relationship between the elapsed time t and the square of the anisotropic gel thickness $X_{2}^{2}$ for *C*_t_ = 0.25 wt.% to compare the experimental result with the theoretical result Equation (36). All data points for all *C*_g_ were on linear curves with positive t-intercepts. The results support the theoretical result Equation (36). The positive t-intercept indicates the presence of “lag time” τ of the orientation dynamics and maintains the orientation dynamics according to the free-energy principle introduced in the previous section. The slope $K{}^{\prime }$ of the t-$X_{2}^{2}$ line increases with increasing *C*_g_. This shows that the threshold $\rho_{f}$ is a decrease function of gelatin concentration. The gelatin concentration dependence of the lag time is not clear in the present experimental data.

For *C*_t_ = 0.05 wt.% and 0.1 wt.%, data covering the entire time range could not be obtained because birefringence could be observed only at late stage. At t≈60 h, birefringence appears across the wide area 0≤x≤3 mm as shown in Figure 3b. Then, the transglutaminase concentration dependences of the slope $K{}^{\prime }$ and of the lag time τ are not clear. 

The dynamics of gelation induced by a contact of polymer solutions with crosslinker solutions has been studied for several types of gelation mechanisms [11,12,42]. One type of gelation (Type I) occurs by inflowing crosslinkers to polymer solutions such as in the gelation of DNA solution by inflowing multivalent cations [29]. Another type of gelation (Type II) occurs by a change of polymer characteristics by inflowing and outflowing low-molecular-weight compounds such as in the gelation of chitosan solution induced by pH change [30,31]. There are cases of gelation induced even by dual mechanisms (Type III) [46], as well as cases accompanied by a transition in mechanisms [29]. The time development of gel layer thickness depends on the mechanism. However, the functional form of the “initial” behavior is common as $X^{2}\propto t$, since the gelation occurs via a diffusion-limited process. The dependence of the proportional constant *K* on the concentration of the constituent molecules is also different depending on the mechanism. *K* is proportional to the concentration of crosslinker molecules and inversely proportional to the crosslinking site on the polymer molecules in Type I. On the other hand, *K* is proportional to the concentration of the inflow molecules and inversely proportional to the outflow molecules, but does not depend on the concentration of the polymer molecules in Type II. In the current case of transglutaminase/protein solution, *K* did not depend on concentrations of any constituent components, as predicted by Equation (21). This theoretical conclusion results from the fact that the enzymatic reaction is much faster than the diffusion of enzymes, and enzymes are repeatedly used for crosslinking reactions assumed in theory.

In the previous systems for Type I and II, the gelation and the orientation appear at the same time, such that both dynamics can be expressed by the same equation. The difference between those systems and the present system can be attributed to the free-energy function $F\left(\Psi \right)$ i.e., the “curvature” at $\Psi =\Psi_{\infty }$, *B*$=\partial^{2}F(\Psi_{\infty })/\partial \Psi^{2}$ in the previous systems might be large. The relaxation time τ is negligibly short since B in the previous systems is quite large. Therefore, the diffusion of crosslinkers could also substantially be the rate-limiting for orientation of protein molecules. Since the threshold value $\rho_{f}$ should be nearly zero, the two slopes K and $K{}^{\prime }$ are the same, and the gelation and orientation appear at the same time. 

### 3.2. Discussion about Free-Energy Function and Orientation Dynamics

Let us consider the free energy F=F(Ψ,ρ) describing the isotropic–anisotropic transition of the gel. The free energy shows that the value of $\Psi =\Psi_{\infty }$ minimizing F changes from zero to finite with increasing transglutaminase concentration ρ. Two types of the change, the first-order and the second-order phase transition type, could exist. 

According to the Landau theory for the phase transition [43], a typical free energy expressing the second-order phase transition is given by

(37) $$F\left(\Psi ,\rho \right)=F_{2nd}\left(\Psi ,\rho \right)=F_{0}+a\left(\rho \right)\Psi +{\frac{1}{2}b}_{0}\Psi^{2},$$

where $b_{0}$ is a positive constant, $F_{0}$ is the free-energy value in the isotropic gel state, and a(ρ) is a function of transglutaminase concentration. A threshold concentration $\rho_{f}$ is present, and a(ρ)≥0 when $\rho \leq \rho_{f}(\phi )$ and $a\left(\rho \right)<0$ when $\rho <\rho_{f}(\phi )$. If we pay attention to only the behaviors near $\rho =\rho_{f}$, we can assume that

(38) $$a\left(\rho \right)=a_{0}\left(\rho_{f}(\phi )-\rho \right),$$

where $a_{0}$ is a positive constant. 

For the first-order phase transition, we have two typical types of the free energy:

(39) $$F\left(\Psi ,\rho \right)=F_{1stA}\left(\Psi ,\rho \right)=F_{0}+a_{1}\Psi +b\left(\rho ,\rho_{f0}(\phi )\right)\Psi^{2}+\frac{1}{3}c_{0}\Psi^{3},$$

and

(40) $$F\left(\Psi ,\rho \right)=F_{1stB}\left(\Psi ,\rho \right)=F_{0}+a\left(\rho ,\rho_{f0}(\phi )\right)\Psi -\frac{1}{2}b_{1}\Psi^{2}+\frac{1}{3}c_{0}\Psi^{3},$$

where $a_{1}$, $b_{1}$ and $c_{0}$ are positive constants. The functions of the transglutaminase concentration ρ, $a\left(\rho ,\rho_{f0}(\phi )\right)$ and $b\left(\rho ,\rho_{f0}(\phi )\right),$ change sign at a transglutaminase concentration $\rho =\rho_{f0}$; $a\left(\rho ,\rho_{f0}(\phi )\right)\geq 0$ and $b\left(\rho ,\rho_{f0}(\phi )\right)\geq 0$ when $\rho \leq \rho_{f0}(\phi )$, and $a\left(\rho ,\rho_{f0}(\phi )\right)<0$ and $b\left(\rho ,\rho_{f0}(\phi )\right)<0$ when $\rho >\rho_{f0}(\phi )$.

The second-order phase-transition-type free energy $F_{2nd}$ is rewritten as

(41) $$F_{2nd}\left(\Psi ,\rho \right)=\frac{1}{2}b_{0}{\left(\Psi +\frac{a}{b_{0}}\right)}^{2}-\frac{1}{2}\frac{a^{2}}{b_{0}}+F_{0}.$$

Then, the time-dependent GL equation corresponding to Equation (24) is written as

(42) $$\frac{\partial \Psi }{\partial t}=-\Gamma b_{0}\left(\Psi -\Psi_{\infty }\right),$$

where

(43) $$\Psi_{\infty }{=\Psi }_{\infty }\left(\phi ,\rho \left(x,t\right)\right)=\left\{\begin{array}{cc} 0 & \left(\rho \left(x,t\right)<\rho_{f}\left(\phi \right)\right)\\ \frac{a_{0}}{b_{0}}(\rho \left(x,t\right)-\rho_{f}\left(\phi \right)) & \left(\rho \left(x,t\right)\geq \rho_{f}\left(\phi \right)\right) \end{array}\right..$$

The initial condition of the equation is given by $\Psi \left(t_{g}\left(x\right)\right)=0$. Then, the solution of Equation (42) is obtained as

(44) $$\Psi \left(x,t\right)=\left\{\begin{array}{cc} 0, & t<t_{a}(\phi ,x)\\ \frac{a_{0}}{b_{0}}e^{-\frac{t}{\tau }}\int_{t_{a}\left(\phi ,x\right)}^{t}\left(\rho \left(x,t^{\prime }\right)-\rho_{f}\left(\phi \right)\right)e^{\frac{t^{\prime }}{\tau }}dt^{\prime }, & t\geq t_{a}(\phi ,x) \end{array}\right.,$$

where

(45) $$\tau =\frac{1}{\Gamma b_{0}}.$$

To estimate the characteristic time of the relaxation behavior, we can evaluate the integrand on the right-hand side as

(46) $$\left(\rho \left(x,t^{\prime }\right)-\rho_{f}\left(\phi \right)\right)e^{\frac{t^{\prime }}{\tau }}≅\left(\rho \left(x,\infty \right)-\rho_{f}\left(\phi \right)\right)e^{\frac{t^{\prime }}{\tau }}=\left(\rho_{s}-\rho_{f}\right)e^{\frac{t^{\prime }}{\tau }}.$$

Then, we have

(47) $$\Psi =\Psi \left(x,t\right)=\left\{\begin{array}{cc} 0, & t<t_{a}(\phi ,x)\\ \Psi_{\infty ,0}\left(1-e^{-\left(t-t_{a}\left(\phi ,x\right)\right)/\tau }\right), & t\geq t_{a}(\phi ,x) \end{array}\right.,$$

where

(48) $$\Psi_{\infty ,0}=\frac{a_{0}(\rho_{s}-\rho_{f})}{b_{0}}.$$

Hence, we have the following gel orientation dynamics being the same as that derived in the previous section:

(49) $$t=t_{\mathrm{ag}}\left(X_{2}^{2}\right)=\frac{1}{K{}^{\prime }}X_{2}^{2}+\tau ,$$

where $K{}^{\prime }$ is given by Equation (35). The expression of τ given by Equation (45) shows that the lag time does not depend on $\rho_{s}$. This result was verified by obtaining the linear relationship between t and $X_{2}^{2}$ for a variety of concentrations of transglutaminase solution, although only the time course of $X_{2}^{2}$ for *C*_t_ = 0.25 wt.% could be obtained, whereas that for *C*_t_ = 0.1 wt.% and *C*_t_ = 0.05 wt.% could not be obtained in the present experiment.

Equation (48) shows that, at low transglutaminase concentration (thus, a small value of $(\rho_{s}-\rho_{f}$)), the “final” order parameter value $\Psi_{\infty ,0}$ is small. This result indicates that the birefringence of the anisotropic gel at lower transglutaminase concentrations is smaller.

The difference in the relaxation behaviors obtained in the previous section shown by Equation (33) and that based on the free energy $F_{2nd}\left(\Psi ,\rho \right)$ shown by Equation (44) appears at the early time of gel orientation. In the time region $t-t_{a}\approx 0$, Equation (44) gives an increase in birefringence with the square of time interval $\Psi \propto {\left(t-t_{a}\right)}^{2},$ whereas Equation (33) gives that with the time interval $\Psi \propto \left(t-t_{a}\right).$ The validity of the free energy $F_{2nd}$ may be verified by observing time evolution of birefringence in the early time range. 

For the free energy $F_{1stA}\left(\Psi ,\rho \right)$, in order that the gel–polymer orientation occurs, a high transglutaminase concentration satisfying the following inequality is required:

(50) $$b<-4\sqrt{\frac{a_{1}c_{0}}{3}}.$$

In the high transglutaminase concentration condition, the free energy $F_{1stA}\left(\Psi ,\rho \right)$ has a local maximum value $F_{\max}$ at

(51) $$\Psi =\Psi_{-}\equiv \frac{-b-\sqrt{b^{2}-4a_{1}c_{0}}}{2c_{0}}.$$

To change the isotropic gel to the anisotropic gel, the free energy induced by thermal fluctuation is required to exceed the energy barrier $\Delta W\equiv F_{\max}-F_{0}$. The nucleation process changes the isotropic gel to the anisotropic gel passing through the energy barrier. Therefore, the orientation process of the gel polymer derived from $F_{1stA}\left(\Psi ,\rho \right)$ is not the relaxation process expressed by Equation (24) but the nucleation process. Hence, $F_{1stA}\left(\Psi ,\rho \right)$ is not suitable as the free energy F in Equation (22).

When a>0, the free energy $F_{1stB}\left(\Psi ,\rho \right)$ has a local maximum as the free energy $F_{1stA}\left(\Psi ,\rho \right)$. The energy barrier vanishes at a=0 (hence, $\rho =\rho_{f0}$). Let us discuss the orientation dynamics based on $F_{1stB}\left(\Psi ,\rho \right)$ when the energy barrier is absent first. The threshold concentration $\rho_{f}(\phi )$ is obtained from the energy barrier-vanishing condition a=0. Hence, $\rho_{f}\left(\phi \right)=\rho_{f0}(\phi )$. When the transglutaminase concentration reaches the threshold concentration $\rho_{f}(\phi )$, the relaxation process from the isotropic gel to the anisotropic gel starts. The onset time $t_{a}(\phi ,x)$ of the relaxation is obtained from $\rho \left(x,t_{a}\right)=\rho_{f0}(\phi )$. Hence, we have the expression equivalent to Equation (32).

(52) $$t_{a}\left(\phi ,x\right)=\frac{1}{{\left(1-\frac{\rho_{f0}\left(\phi \right)}{\rho_{s}}\right)}^{2}}t_{g}\left(x\right).$$

The equation expressing the relaxation behavior based on $F_{1stB}\left(\Psi ,\rho \right)$ is given by

(53) $$\frac{\partial \Psi }{\partial t}=-\Gamma \kappa \left(\Psi -\Psi_{\infty }\right),$$

where

(54) $$\Psi_{\infty }=\frac{b_{1}+\sqrt{b_{1}^{2}-4a(\rho \left(x,t\right),\rho_{f0})c_{0}}}{2c_{0}},$$

and

(55) $$\kappa =\sqrt{b_{1}^{2}-4a_{\infty }c_{0}},$$

with

(56) $$a_{\infty }=a\left(\rho {|}_{t\to \infty },\rho_{f0}\right)=a\left(\rho_{s},\rho_{f0}\right).$$

The initial condition of the equation is given by $\Psi \left(t_{a}\left(\phi ,x\right)\right)=0$. Approximating $\Psi_{\infty }$ by $\Psi_{\infty ,0}\equiv (b_{1}+\sqrt{b_{1}^{2}-4a_{\infty }c_{0}})/(2c_{0})$, we can obtain the solution of the equation as

(57) $$\Psi =\Psi \left(x,t\right)=\left\{\begin{array}{cc} 0, & t<t_{a}\left(\phi ,x\right)\\ \Psi_{\infty ,0}\left(1-e^{-\left(t-t_{a}\left(\phi ,x\right)\right)/\tau }\right), & t\geq t_{a}\left(\phi ,x\right) \end{array}\right.,$$

where

(58) $$\tau =\frac{1}{\Gamma \kappa }.$$

Hence, the gel orientation dynamics based on $F_{1stB}$ is given by

(59) $$t=t_{\mathrm{ag}}\left(X_{2}^{2}\right)=\frac{1}{K{}^{\prime }}X_{2}^{2}+\tau ,$$

with $K^{\prime }={\left(1-\rho_{f0}/\rho_{s}\right)}^{2}K$. Note that, since κ depends on $\rho_{s}$, the lag time τ depends on $\rho_{s},$ in contrast to the gel orientation dynamics based on $F_{2nd}\left(\Psi ,\rho \right)$. In the low-concentration limit of transglutaminase solution, $\rho_{s}=\rho_{f}(\phi )$, $\Psi_{\infty ,0}$ does not vanish. Hence, the birefringence of the anisotropic gel does not strongly depend on the concentration of the transglutaminase solution and is not so small at low concentration condition $\rho_{s}\approx \rho_{f}(\phi )$.

Next, let us discuss the orientation dynamics based on $F_{1stB}\left(\Psi ,\rho \right)$ when the energy barrier is present. The isotropic gel changes to the anisotropic gel through the nucleation process when the following inequality is satisfied:

(60) $$F_{1stB}\left(\Psi_{\infty },\rho \right)\leq F_{0}.$$

From the inequality, the range of a in which the isotropic–anisotropic transition occurs through the nucleation process is given by

(61) $$0\leq a\leq \frac{3b_{1}^{2}}{16c_{0}}.$$

Adopting the linear approximation for a as $a\left(\rho ,\rho_{f0}\right)=a_{0}\left(\rho_{f0}(\phi )-\rho \right)$, we obtain the condition for the orientation dynamics induced by the nucleation process as

(62) $$\rho_{f0}-\frac{3b_{1}^{2}}{16c_{0}}\leq \rho \leq \rho_{f0}.$$

The above discussion based on $F_{1stB}$ leads to the prediction that the mechanism of the isotropic–anisotropic transition changes depending on the concentration of transglutaminase solution; the orientation dynamics is derived by the relaxation process for a high-concentration transglutaminase solution ($\rho_{s}>\rho_{f0}$) and by the nucleation process for a low concentration ($\rho_{f0}-3b_{1}^{2}/(16c_{0})\leq \rho \leq \rho_{f0}$). At quite low concentrations $\rho_{s}<\rho_{f0}-3b_{1}^{2}/(16c_{0})$, an anisotropic gel does not appear. The experimental result for Series 2 may be explained by the idea of the mechanism change; for *C*_t_ = 0.05 and 0.1 wt.%, the gel–polymer orientation proceeds via the nucleation process, while, for 0.25 wt.%, it proceeds via the relaxation process. Among the three types of free energy discussed in the present section, the free-energy function $F_{1stB}\left(\Psi_{\infty },\rho \right)$ seems to best explain the present experimental results. However, more precise experimental data will be required to determine the definitive free energy function form.

The present analysis could be modified to apply to multicomponent crosslinking reactions. For example, it is interesting to consider the case when gelation is induced by both catalysts such as enzymes and crosslinkers, where the reaction rate does not depend on enzyme concentration but increases with crosslinker concentration. It is also interesting to modify the present theory to the competitive reaction of enzymes and inhibitors, where the enzymatic reaction rate does not depend on the enzyme concentration, but the inhibition reaction rate increases with inhibitor concentration above a threshold which does not depend on enzyme concentration. On the other hand, it is difficult to apply the present theory directly to the blood coagulation cascade reaction where many enzymes are used for reactions activating enzyme precursors called zymogens to activate the next step of the enzymatic reaction, but the activated precursors (active enzymes) are transformed to the inactive form immediately.

## 4. Conclusions

In summary, we studied the dynamics of gelation of gelatin solution induced by diffusion of transglutaminase both experimentally and theoretically. Experimentally, the relationship between the thickness of the yielded gel in the tube and the elapsed time was measured for various concentrations of gelatin and transglutaminase. The isotropic gelation was observed and followed by gel polymer orientation after a lag time. The time course of the isotropic gel growth was simply expressed by a proportional relation between the elapsed time and the square of the isotropic gel thickness independent of gelatin and transglutaminase concentrations. For the anisotropic gel, the elapsed time was a linear function of the square of the gel thickness with a lag time. Using the moving boundary picture and the GL theory, we theoretically approached the isotropic and anisotropic gel growth and derived the equations expressing those growth behaviors. We showed that the experimental result is well explained by the theory. We also discussed the free-energy function form expressing isotropic–anisotropic gel transition. 

The fact that the anisotropy appears after the lag time shows that the rate-limiting process of the isotropic gelation dynamics of the gelatin solution and that of the orientation dynamics of the gelatin gel are different. Since the orientation dynamics is observed on a macroscopic space–time scale, the orientation process was expected to be a relaxation phenomenon driven by thermodynamic forces. Since the orientation occurs in an isothermal condition, the driving force is the difference in free energy between the isotropic and anisotropic gel phases. Hence, the orientation dynamics is free-energy-limited. In an isolated system, the driving force is entropy; however, since the experimental system discussed in this article is an isothermal system, the driving force is free energy. We introduced the order parameter (degree of orientation) Ψ as a thermodynamic variable that distinguishes the isotropic state and the anisotropic state. The free energy under fixed value Ψ of the degree of orientation was written as F(Ψ). The value of the order parameter after enough time has elapsed was denoted as $\Psi_{\infty }$. The enzyme influx first forms an isotropic gel (Ψ=0); then, the gelatin gel relaxes from an isotropic structure with Ψ=0 to an anisotropic structure with $\Psi =\Psi_{\infty }>0$ according to the second law of thermodynamics because of $F\left(0\right)>F(\Psi_{\infty })$. 

The GL Equation (22) has been used with much success as a general method to describe relaxation processes. Therefore, we used the GL equation in the present article. The experimental results presented in this article do not directly provide information on the microscopic structure of the anisotropic gel and mechanism of anisotropy. Therefore, the free energy cannot be derived on the basis of the microscopic structure of the gelatin gel. In the present article, a candidate for the functional form F(Ψ) was explored. This is because the function form of the free energy is an important clue to deduce the structure of the anisotropic gel and the microscopic mechanism of anisotropy.

## 5. Materials and Methods

Porcine gelatin (APH-150) was a gift from Nitta Gelatin Inc. The gelatin sample consisted of mixtures of units of collagen, each having molar mass of 95 kDa, together with their oligomers and breakdown polypeptides. Microbial transglutaminase triturated by dextrin (TG), a gift from Ajinomoto Co., Inc. (Tokyo, Japan), had a molar mass of 38 kDa, which is much smaller than that of gelatin. Reagent-grade HCl, NaCl, and Tris(hydroxymethyl)aminomethane were purchased from Wako Pure Chemical Ind. Ltd. (Osaka, Japan) MilliQ water was used to prepare a buffer solution at pH = 7 by adding 10 mM Tris-HCl and 40 mM NaCl. Gelatin solutions and transglutaminase solutions with various concentrations were prepared by using the buffer solution. Then, 1.5 mL of gelatin solution was poured into eight glass tubes with 9 mm inner diameter capped at the bottom. The glass tubes were incubated at 5 °C in a refrigerator for 1 h to obtain a physical gel having a solid surface. Then, 2.5 mL of TG solution was put on the gel in the glass tube, and capped at the top. The glass tubes were settled in a water bath with the temperature controlled at 37.0 ± 0.2 °C, where the gelatin gel returned to a gelatin solution and transglutaminases catalyzed the formation of isopeptide bonds between γ-carboxamide groups of glutamine residue and ε-amino groups of lysine residue intermolecularly to make cross-bridges. Roughly every 12 h, one of eight tubes was taken from the water bath, and the top and bottom caps were removed to extract the gel portion. The volume of the gel was determined by putting it into a water column and measuring the change in water height in the column. The average thickness of the gel layer *X*_1_ was calculated from the volume and the inner diameter of the glass tube. A part of the gel layer had birefringence. A photograph of the glass tube was taken also under crossed nicols just before measuring the gel volume as above. The average anisotropic (birefringent) gel layer thickness *X*_2_ was determined from the projection area of the birefringent layer by assuming cylindrical symmetry. Two series of experiments were conducted: Series 1 at constant TG concentration *C*_t_ = 0.25 wt.% and various gelatin concentrations *C*_g_ = 10, 15, and 25 wt.%; Series 2 at constant gelatin concentration *C*_g_ = 20 wt.% and various TG concentrations *C*_t_ = 0.05, 0.1, and 0.25 wt.%.

## Data Availability

Not applicable.
